# Phytochemical differences of hemp (*Cannabis sativa* L.) leaves from different germplasms and their regulatory effects on lipopolysaccharide-induced inflammation in Matin-Darby canine kidney cell lines

**DOI:** 10.3389/fnut.2022.902625

**Published:** 2022-07-22

**Authors:** Yi Liu, Ai-Ping Xiao, Hao Cheng, Liang-Liang Liu, Kin Weng Kong, Hong-Yan Liu, Ding-Tao Wu, Hua-Bin Li, Ren-You Gan

**Affiliations:** ^1^Research Center for Plants and Human Health, Institute of Urban Agriculture, Chengdu National Agricultural Science and Technology Center, Chinese Academy of Agricultural Sciences, Chengdu, China; ^2^Institute of Bast Fiber Crops, Chinese Academy of Agricultural Sciences, Changsha, China; ^3^Department of Molecular Medicine, Faculty of Medicine, Universiti Malaya, Kuala Lumpur, Malaysia; ^4^Key Laboratory of Coarse Cereal Processing (Ministry of Agriculture and Rural Affairs), Sichuan Engineering and Technology Research Center of Coarse Cereal Industrialization, School of Food and Biological Engineering, Chengdu University, Chengdu, China; ^5^Guangdong Provincial Key Laboratory of Food, Nutrition and Health, Department of Nutrition, School of Public Health, Sun Yat-sen University, Guangzhou, China

**Keywords:** hemp germplasm, hemp leaves, phytochemicals, anti-inflammatory effects, Matin-Darby canine kidney cells

## Abstract

The increasing demand of hemp (*Cannabis sativa* L.) has attracted more interest in exploring its phytochemical profile and bioactivities, such as anti-inflammatory effect. In this study, the phytochemicals of different hemp leaves were investigated, with the content order: total saponins content (TSC) > total alkaloids content (TAC) > total phenolics content (TPC) > total flavonoids content (TFC) > cannabinoids. Hemp leaves from Shanxi accumulated higher flavonoids and cannabinoids (i.e., THC, CBD, and CBN), while phenolics were more abundant in those from Hunan. A lipopolysaccharide (LPS)-induced inflammatory Matin-Darby canine kidney (MDCK) cell model was established to evaluate the anti-inflammatory effects of hemp leaf extracts. Hemp leaf extracts, especially the D129 and c7, significantly increased cell viability of LPS-induced inflammatory MDCK cells, and D132 significantly decreased the secretion of pro-inflammatory cytokines (TNF-α and IL-6) and the lactate dehydrogenase (LDH) activity. Except for c12, other hemp leaf extracts obviously decreased the cell morphological damage of LPS-induced inflammatory MDCK cells. The correlation analysis revealed that cannabinol (CBN) and TPC showed the strongest correlation with anti-inflammatory activities, and hierarchical clustering analysis also showed that hemp germplasms from Shanxi might be good alternatives to the common cultivar Ym7 due to their better anti-inflammatory activities. These results indicated that hemp leaves were effective in LPS-induced inflammatory MDCK cells, and flavonoids and cannabinoids were potential geographical markers for distinguishing them, which can provide new insights into the anti-inflammatory effect of hemp leaves and facilitate the application of hemp leaves as functional ingredients against inflammatory-related disorders.

## Introduction

Hemp (*Cannabis sativa* L.) plant is a multipurpose industrial crop widely cultivated in the world (1). In recent years, the application of various parts of hemp plant, such as hemp inflorescence, hempseed oil, and hemp sprouts, has represented a valuable source of nutraceuticals and functional foods (2, 3). While hemp leaves are usually consumed as a raw food, like juices and salads (2). A vast array of phytochemicals including cannabinoids, flavonoids, phenolics, alkaloids, and saponins has been successfully isolated from hemp (4–7). Phytochemicals are responsible for diverse pharmacological functions. In particular, hemp leaves contain more abundant phytochemicals than other parts of hemp, because the phytochemicals in the hemp plant chronologically decrease from flowers, leaves, stems, and seeds to the roots (8, 9). Hemp leaves are regarded as a universal material of great practical and economic value due to their diverse pharmacological functions, such as antioxidant, anti-inflammatory, and hypoglycemia effects (10).

Inflammation is a normal process of host defense and tissue healing, but excessive or unresolved inflammation can lead to many health disorders (9, 11, 12). As a potential supplement of the nutraceuticals and functional foods, hemp leaves may have an overall positive effect on inflammation. However, the details about the anti-inflammatory properties of hemp leaves and their protective mechanism are scanty. Thus, there is an increasing interest in exploring the anti-inflammatory activities of hemp leaves. There are different hemp leaves available from different major cultivating regions in China, which may vary in phytochemicals (cannabinoids, flavonoids, phenolics, alkaloids, and saponins) as well as anti-inflammatory properties. Therefore, it is necessary to compare and identify potential hemp leaves rich in bioactive components and exhibiting good anti-inflammatory activities.

A previous study reported that flavonoid variation in hemp leaves has the potential as a geographical marker (13). Therefore, it is speculated that hemp leaves from different regions can be distinguished by comparing their phytochemicals. In this study, it is aimed to compare the phytochemical profiles of hemp leaves from 12 germplasms in 2 regions (Shanxi and Hunan) of China, and to further test their anti-inflammatory activities in LPS-induced Matin-Darby canine kidney (MDCK) cells. This study can provide a better understanding of the relationships between hemp phytochemicals and anti-inflammatory activities of hemp leaves, which can have potential use as novel alternative sources for food, cosmetic, and medical ingredients.

## Materials and methods

### Materials and reagents

Hemp leaves of different germplasms, “D129, D130, D132, D134, D142, D361” cultivated in Fenyang, Shanxi Province and “Yunma7 (Ym7), c2, c4, c7, c8, c12” cultivated in Changsha, Hunan Province, were provided by the Institute of Bast Fiber Crops, Chinese Academy of Agricultural Sciences, China, which were collected before flowering by the local police in 2019. Cell Counting Kit-8 (CCK-8; CK04), Rayson-jimsa staining solution (Biosharp, BL880A), and lipopolysaccharide (S11060-10 mg) were purchased from Yuanye Bio-Technology Co., Ltd. (Shanghai, China). Canine TNF alpha ELISA Kit (Mm-36988O2), Canine IL-6 ELISA Kit (MM-1546O2), and LDH Cytotoxicity Assay Kit (Cominbio, LDH-1-Y) were purchased from Zhongchuang Hongda Technology Co., Ltd. (Beijing, China). Standards of cannabidiol (CBD), tetrahydrocannabinol (THC), cannabinol (CBN), rutin, gallic acid, tea saponins (98%), and colchicine were purchased from Yuanye Bio-Technology Co., Ltd. (Shanghai, China). All other reagents were purchased from Sinopharm Chemical Reagent Co., Ltd. (Shanghai, China) and were of analytical grade unless otherwise stated. Ultrapure water (18.2 MΩ cm, resistivity) was obtained from an ELGA water purification system (Veolia Water Company, Frankfurt, Germany).

### Heat reflux extraction of hemp leaf

Hemp leaves were dried in a drying oven (BPG-9140A, Shanghai, China) at 45°C for 48 h, and the dried samples were crushed to powders by a pulverizer (KY15-19, Tianjin, China) before passing through an 80-mesh sieve. The powders were preserved at 4°C and extracted by the heat-reflux-extract method according to a previous study with some modifications (14). The extracts were prepared by mixing 1 g of each sample with 20 mL of 70% aqueous ethanol solution in a thermostat water bath (WEB-6, DAIHAN Scientific Co., Ltd., Korea) at 80°C for 3 h. All extracts were centrifuged and the supernatants were stored at 4°C for later use.

### Phytochemical analysis

#### HPLC quantification of main cannabinoids

CBD, THC, and CBN are leading cannabinoids in hemp leaves. Therefore, their contents in hemp leaves were quantified using HPLC following a previous method with slight modifications (15). The HPLC analysis was conducted using an Agilent HPLC-1260 system with a Thermo Hypersil GOLD-C18 column (4.6 × 100 mm, 5 μm) thermostated at 25°C. Gradient elution was used with solvent A as 0.1% aqueous acetic acid solution (v/v) and solvent B as 100% acetonitrile. The gradient elution was as follows: 0–10 min, 1–62% B. The flow rate was set at 0.5 mL/min with the sample injection volume of 1 μL and the detection wavelength was set at 220 nm. The cannabinoids were quantified using their respective calibration curves prepared from the standards at different concentrations: CBD (10–100 μg/mL), THC (10–50 μg/mL), and CBN (10–100 μg/mL). The results were expressed as the percentage of yields (mg/100 g) of these compounds from the hemp leaf powders. The lower limit of detection (LOD) of CBD, THC, and CBN were 0.03, 0.06, and 0.02 μg/mL, respectively. The quantification (LOQ) of CBD, THC, and CBN were 0.11, 0.20, and 0.079 μg/mL, respectively.

#### Total phenolic content

Total phenolic content (TPC) in the hemp leaf extracts was determined using the Folin-Ciocalteu method described previously with a little modification (16). A 20 μL of hemp leaf extract (0.2 mg/mL) diluted from the supernatant mentioned in section “Heat reflux extraction of hemp leaf” was mixed with 1,980 μL of water and subsequently with 200 μL of Folin-Ciocalteu reagent. After 5 min incubation, 0.8 mL of sodium carbonate (10%, w/v) was added and allowed to incubate in dark for 60 min at room temperature. The absorbance was measured at 765 nm using a UV-vis spectrophotometer (UV2700, Shimadzu, Kyoto, Japan). TPC was quantified using a calibration curve of gallic acid (0-20 μg/mL). The results were expressed as mg gallic acid equivalents/g of hemp leaf powders (mg GAE/g).

#### Total flavonoid content

Total flavonoid content (TFC) was determined according to the method described by Bajalan et al. (17). The extract (1 mL at 0.2 mg/mL) was mixed with 2 mL of AlCl_3_ solution (0.1 M). After 6 min of incubation, 3 mL of potassium acetate solution (0.1 M) was then added to the mixture before incubating at room temperature for another 6 min. The absorbance was measured at 420 nm using a UV-vis spectrophotometer. The TFC was quantified using a calibration curve of rutin (5-90 μg/mL), and the results were expressed as mg rutin equivalents/g of hemp leaf powders (mg RE/g).

#### Total saponin content

The determination of TSC was performed according to a previous method with minor modifications (18). Briefly, 0.2 mL of vanillin-glacial acetic acid solution (5%, v/v) was added to 0.2 mL of extract (0.2 mg/mL). Then, the mixture was added with 0.8 mL of perchloric acid and heated at 70°C for 15 min. After heating, the mixture was immediately placed in ice water for 15 min to stop the reaction. The final volume was made up to 5 mL with glacial acetic acid. The absorbance of the reaction mixture was measured at 545 nm. The TSC was quantified using a calibration curve of tea saponin (0-120 μg/mL). The results were expressed as mg tea saponin equivalents/g of hemp leaf powders (mg TSE/g).

#### Total alkaloid content

Total alkaloid content (TAC) was determined using the Dragendorff precipitation assay (19). After removing the solvent from the 1 mL extract, 1 mL of HCl (2 M) was used to dissolve the extract. The sample was transferred to a separatory funnel before adding with 5 mL of 0.1% bromocresol green aqueous solution and 5 mL of phosphate buffer. The mixture was then mixed with 4 mL of chloroform by vigorous shaking twice. The chloroform layer was collected in a volumetric flask and made up to 10 mL with chloroform. The absorbance of the reaction mixture was determined at 470 nm using a UV-vis spectrophotometer. The TAC was quantified using a calibration curve of colchicine (0-70 μg/mL), and the results were expressed as mg colchicine equivalents/g of hemp leaf powders (mg CE/g).

### Anti-inflammatory effects of hemp leaf extracts on lipopolysaccharide-induced Matin-Darby canine kidney cells

#### Cell culture

MDCK cell line was kindly provided by the Institute of Animal Sciences, Chinese Academy of Agricultural Sciences (Beijing, China). MDCK cells were cultured in a T25 cell culture flask at 37°C in 5% CO_2_ for 24 h in Dulbecco’s Modified Eagle’s Medium (DMEM)/F12 supplemented with 5% fetal bovine serum (FBS). Then, the medium was removed and the cells were washed with 1 mL PBS. The PBS was removed and the MDCK cells were treated with 1 mL of trypsin (100 U) for about 6 min in the 5% CO_2_ incubator until all cells were detached from the culture flask. After trypsinization, cells were suspended with a medium supplemented with 5% serum to prepare a cell suspension.

#### Cellular viability assay

Cell counting kit-8 (CCK-8) assay is a classic test for evaluating cellular activity (20). It is performed by utilizing 2-[2-methoxy-4-nitrophenyl]-3-[4-nitrophenyl]-5-[2,4-disulfophenyl]-2H-tetrazolium (WST-8) reduced by dehydrogenases in cells to give formazan soluble in culture medium, and the amount of the formazan dye is directly proportional to the number of living cells (21). The effect of hemp leaf extracts on cellular activity was measured according to the instruction of the CCK-8 assay. Ym7 hemp leaf extract was used to determine a suitable concentration of hemp leaf extracts on MDCK cells. MDCK cell suspension (1 × 10^5^ cells per well) from section “Cell culture” was plated in 96-well plates and incubated at 37°C in 5% CO_2_. After cell confluency reached 80%, cells of each well were washed with PBS before being added with 100 μL of medium with 2% FBS and 10 μL of Ym7 hemp leaf extract (0.005, 0.05, 0.5, and 5 mg/mL), and then incubated for 24 h at 37°C in 5% CO_2_. A 10 μL CCK-8 was added to each well and incubated for 4 h at 37°C in 5% CO_2_. Culture supernatants were collected to measure the viability of cells.

The effect of LPS on cellular activity was also measured using CCK-8 assay to determine the optimal LPS concentration for inducing cellular inflammatory damage. Briefly, MDCK cells (1 × 10^5^ cells per well) from section “Cell culture” were seeded into 96-well plates and allowed to incubate at 37°C in 5% CO_2_ for 4 h. Two hours before LPS intervention, serum-free medium was used to continue culturing for another 2 h. Like the above cellular activity assay for Ym7, cells were then plated and treated for 24 h with LPS (10, 50, 100, and 200 μg/L). Culture supernatants were collected to measure the viability of cells. The optical density (OD) was measured by a microplate reader (Epoch, Biotek, United States) at 450 nm.

Cellsurvivalrate%=(A-SA)B/(A-CA)B×100%.

Where, A_*S*_ is the absorbance of treatment (cells + medium + CCK-8 + LPS/hemp leaf extracts), A_*C*_ is the absorbance of control (cells + medium + CCK-8 + water), A_*B*_ is the absorbance of blank (medium + CCK-8). Curves were depicted with concentrations as the abscissa and the survival rate of MDCK cells as the ordinate. Each experiment was conducted in triplicate.

#### Different treatments on lipopolysaccharide-induced inflammatory Matin-Darby canine kidney cells

LPS aqueous solution (200 μg/mL) that induced the highest cellular inflammatory damage and hemp leaf extract (50 μg/mL, diluted with water) with no evident growth inhibitory activity on MDCK cells were chosen for the subsequent experiments. A 100 μL MDCK cells (1 × 10^5^ cells) were seeded into 96-well plates for 4 h, and the medium was removed and replaced with four different treatments for 24 h: (a) control group (water), (b) LPS aqueous solution (200 μg/mL), (c) 12 hemp leaf extracts (50 μg/mL) + LPS aqueous solutions (200 μg/mL), and (d) 12 hemp leaf extracts (50 μg/mL), with each treatment conducted in triplicate. Culture supernatants from the basolateral side were collected for the measurement of cell viability, the contents of lactate dehydrogenase (LDH), and the levels of tumor necrosis factor-alpha (TNF-α) and interleukin 6 (IL-6). Subsequently, the MDCK cells were collected for morphology observation.

#### Wright-Giemsa staining of Matin-Darby canine kidney cells

The Wright-Giemsa stain, a modified Romanowsky stain, is a combination of basic dyes (methylene blue and its oxidative products, azure A and azure B) and acidic dye (eosin) (19). The effect of hemp leaf extracts on the morphology of MDCK cells was studied by the Wright-Giemsa staining. The MDCK cells of different treatments from section “Different treatments on lipopolysaccharide-induced inflammatory Matin-Darby canine kidney cells” were collected and centrifuged at 1,200 × g for 5 min. Then, 100 μL of sterile PBS was added to resuspend the cells. After that, three drops of Wright stain were added and incubated for three min. The same amount of Giemsa stain was then added and allowed to stain for 10 min. The cell morphology was observed under an inverted microscope after the staining.

#### Lactate dehydrogenase cytotoxicity assay

The membrane integrity of MDCK cells was measured by the LDH cytotoxicity assay kit. The culture supernatants of different treatments were prepared and collected as described in section “Different treatments on lipopolysaccharide-induced inflammatory Matin-Darby canine kidney cells” The culture supernatants were centrifuged at 6,600 × g for 10 min. The LDH activity in the culture supernatant was determined strictly following the instructions of the LDH detection kit. The assay was conducted in triplicate.

#### Cytokine measurements using enzyme-linked immunosorbent assay

The expression of TNF-α and IL-6 were measured by the commercial enzyme-linked immunosorbent assay (ELISA) kits, according to the manufacturer’s protocol. The culture supernatants of different treatments were collected as described in section “Different treatments on lipopolysaccharide-induced inflammatory Matin-Darby canine kidney cells” and centrifuged at 1,200 × g for 15 min. After the color reaction was terminated, the OD value of each well at 450 nm was measured by a microplate reader, and the concentrations of TNF-α and IL-6 in the culture supernatants were calculated based on the standard curve. The assay was conducted in triplicate.

### Statistical analysis

Results were expressed as means ± standard deviations (SD). One-way analysis of variance (ANOVA) coupled with *post hoc* Least Significant Difference (LSD) and Duncan tests were performed using IMB SPSS 20.0 software (IBM Corp., Armonk, NY, United States). Statistical significance was defined at *p* < 0.05. The heatmap was generated by OriginPro 2021b (OriginLab, Northampton, MA, United States).

## Results and discussion

### Phytochemical analysis of different hemp leaves

Colorimetric methods and HPLC are the convenient and important first-line methods for phytochemical analysis. In this study, HPLC analysis was accomplished within 12 min. The typical HPLC chromatogram showed peaks of CBD, THC, and CBN with satisfied separation at 3.39, 6.77, and 9.66 min, respectively (Supplementary Figure 2). The contents of CBD, THC, and CBN in 12 hemp leaf extracts (6 germplasms from Shanxi Province and 6 germplasms from Hunan Province) are depicted in Table 1. Usually, *C. sativa* can be mainly divided into two types according to the content of THC, including the drug-type (commonly called cannabis, with THC > 300 mg/100 g) and the fiber-type (commonly called hemp, with THC < 300 mg/100 g) (6, 22, 23). In the current study, the THC in the 12 hemp leaf extracts was lower than 300 mg/100 g, especially THC in the germplasms c2, c4, c7, and c8, which were below the detection limit. Hence, it is confirmed that these 12 hemp germplasms are the industrial hemp that could be cultivated and utilized. CBD and CBN, known as non-psychoactive cannabinoids, are commonly found in hemp that has shown anti-inflammatory and analgesic effects (24). Recently, anti-inflammatory effects of CBD have been demonstrated in various preclinical models, and CBD presenting in the Δ^9^-THC environment can reduce undesirable effects of Δ^9^-THC, therefore, improves its safety profile (25). The current study found that the contents of CBD and CBN in 12 hemp leaf extracts were different, D134 had the highest level of CBD (281.5 ± 6.0 mg/100 g), and D132 had the highest content of CBN (16.5 ± 0.1 mg/100 g). Additionally, the contents of CBD in c7 and c12, and CBN in Ym7, c2, c4, and c12 were below the detection limit. Overall, the contents of CBD, THC, and CBN were more abundant in hemp leaves from Shanxi, indicating that cannabinoids (i.e., THC, CBD, and CBN) in hemp leaves are potential geographical markers.

**TABLE 1:** The content of cannabinoids, flavonoids, phenolics, saponins, and alkaloids in hemp leaves from Shanxi and Hunan of China.

Samples	CBD	THC	CBN	TFC	TPC	TSC	TAC
	
	(mg/100 g)	(mg/100 g)	(mg/100 g)	(mg RE/g)	(mg GAE/g)	(mg TSE/g)	(mg CE/g)
D129	215.6 ± 5.2^c^	79.1 ± 2.2^f^	11.3 ± 0.14^ab^	5.21 ± 0.26^a^	16.44 ± 0.29^f^	101.20 ± 2.14^a^	37.10 ± 1.65^a^
D130	214.5 ± 2.3^c^	195.3 ± 1.4^c^	14.5 ± 0.2^a^	3.46 ± 0.20^b^	12.66 ± 0.47^i^	87.40 ± 1.79^c^	11.33 ± 0.76^ef^
D132	141.5 ± 3.1^d^	257.9 ± 1.5^b^	16.5 ± 0.10^a^	2.17 ± 0.16^de^	14.96 ± 0.26^gh^	84.58 ± 2.24^d^	13.96 ± 0.59^de^
D134	281.5 ± 6.0^a^	171.9 ± 2.2^d^	8.7 ± 0.08^ab^	4.86 ± 0.09^b^	13.15 ± 0.61^hi^	99.46 ± 0.95^b^	14.28 ± 0.95^de^
D142	261.8 ± 6.9^b^	157.0 ± 14^e^	10.6 ± 0.11^abc^	4.45 ± 0.31^b^	12.02 ± 0.24^i^	107.16 ± 2.72^a^	12.58 ± 0.96^e^
D361	11.5 ± 0.11^g^	285.3 ± 13^a^	9.9 ± 0.05^ab^	4.17 ± 0.19^b^	15.94 ± 0.23^g^	105.14 ± 3.47^a^	11.23 ± 0.67^f^
Ym7	117.5 ± 2.1^d^	32.6 ± 0.09^g^	ND	1.37 ± 0.20^f^	39.49 ± 0.38^a^	54.27 ± 1.99^f^	14.85 ± 0.42^d^
c2	118.1 ± 5^d^	ND	ND	3.07 ± 0.15^c^	32.49 ± 0.06^d^	53.98 ± 3.52^fg^	13.91 ± 0.44^de^
c4	68.3 ± 4^f^	ND	ND	2.45 ± 0.26^d^	37.40 ± 0.24^b^	64.60 ± 3.64^e^	26.73 ± 0.57^a^
c7	ND	ND	5.0 ± 0.07^c^	1.33 ± 0.13^f^	35.72 ± 0.47^c^	104.68 ± 2.73^a^	36.12 ± 0.34^a^
c8	89.8 ± 2.3^e^	ND	3.9 ± 0.06^c^	2.34 ± 0.22^d^	36.64 ± 0.26^bc^	62.44 ± 2.82^e^	28.27 ± 0.83^b^
c12	ND	ND	ND	1.59 ± 0.18^ef^	30.28 ± 0.34^e^	52.74 ± 2.43^f^	25.63 ± 0.72^c^

ND, not detectable; CBD, cannabidiol; THC, tetrahydrocannabinol; CBN, cannabinol; TFC, total flavonoids content; TPC, total phenolics content; TSC, total saponins content; TAC, total alkaloids content. The superscript letters (a–i) for the same column indicated a statistical significance at p < 0.05.

Other than cannabinoids, phenolics, flavonoids, saponins, and alkaloids in the hemp can also serve as important candidates for the biological effects of hemp (24). The contents of TPC, TFC, saponins, and alkaloids in the 12 hemp leaf extracts were found to have great differences, as shown in Table 1. Generally, their contents in hemp leaves are in the descending order as follows: TSC > TAC > TPC > TFC. TSC values ranged from 52.74 ± 2.43 (c12) to 107.16 ± 2.14 (D142) mg TSE/g. TAC values ranged from 11.23 ± 0.67 (D361) to 37.10 ± 1.65 (D129) mg CE/g. TPC values ranged from 12.02 ± 0.24 (D142) to 39.49 ± 0.38 (Ym7) mg GAE/g. TFC values ranged from 1.33 ± 0.13 (c7) to 5.21 ± 0.26 (D129) mg RE/g. In addition, the highest contents of TPC, TFC, saponins, and alkaloids were found in Ym7, D129, D142, and D129, respectively. Hemp leaves from Shanxi accumulated higher TFC, while TPC were more abundant in those from Hunan, indicating that TFC in hemp leaves are also potential geographical marker like a previous study (13). Overall, our results indicated that flavonoids and cannabinoids were potential geographical markers of hemp leaves from different regions. In the meantime, hemp leaves with high levels of cannabinoids, flavonoids, phenolics, saponins, and alkaloids can be potential sources for the development of feeding materials for animals as well as nutraceuticals, cosmetics, and pharmaceuticals for humans.

### The effects of hemp leaf extracts on cellular activity

So far, no studies have focused on the effect of hemp leaf extracts on cellular activity of MDCK cells. In recent years, hemp variety Ym7 has been widely cultivated for hemp fiber production. It is the main cultivar in Yunnan Province, one of the main production areas of hemp (23). Thus, the cellular activity of this cultivar was first evaluated using the CCK-8 assay to identify a suitable concentration of hemp leaf extracts to be treated on MDCK cells.

**FIGURE 1:**
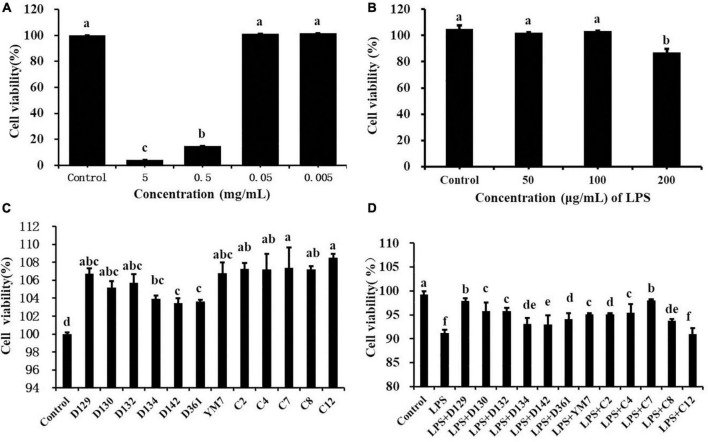
The cytotoxicity effect of hemp leaf extracts on MDCK cells. **(A)** The cytotoxicity of Ym7 hemp leaf extract on MDCK cells. **(B)** Dose-response effect of LPS in causing inflammatory damage to MDCK cells. **(C)** The effect of 12 hemp leaf extracts (50 μg/mL) on the proliferation of MDCK cells. **(D)** The protective effect of hemp leaf extracts (50 μg/mL) against LPS-induced inflammatory damage in MDCK cells. The superscript letters (a–f) indicated a statistical significance at *p* < 0.05.

As shown in Figure 1A and Supplementary Figure 1, different concentrations of hemp leaf extract (0.005, 0.05, 0.5, and 5 mg/mL) exhibited different effects on the viability of MDCK cells. When the concentrations of Ym7 were 0.05 and 0.005 mg/mL, the cell survival rate was 101.36 ± 0.32% and 101.78 ± 0.16%, respectively, close to the control (100%), indicating that the extract had no inhibition of the viability of MDCK cells at lower concentrations. When MDCK cells were treated with Ym7 at 5 and 0.5 mg/mL, the cell viabilities were very low (4.30 ± 0.14% and 15.02 ± 0.23%, respectively), indicating that higher concentrations of hemp leaf extracts had significant inhibition of the viability of MDCK cells. However, the concentration of hemp leaf extract at 0.05 mg/mL or a lower does did not show any inhibition of the viability of MDCK cells. Hence, 0.05 mg/mL was selected for the following experiments to compare the influences of 12 hemp leaf extracts on the viability of MDCK cells.

As shown in Figure 1C, the viability of cells treated by 12 hemp leaf extracts at 0.05 mg/mL were, 104.01 ± 0.06% (D129), 106.71 ± 0.16% (D130), 105.20 ± 0.24% (D132), 105.80 ± 0.19% (D134), 103.95 ± 0.16% (D142), 103.44 ± 0.26% (D361), 103.62 ± 0.21% (Ym7), 106.79 ± 0.26% (c2), 107.30 ± 0.26% (c4), 107.40 ± 0.26% (c7), 107.208 ± 0.26% (c8), and 108.50 ± 0.26% (c12), higher than the control, indicating that the hemp leaf extracts enhanced the viability of MDCK cells at 0.05 mg/mL.

### The protective effects of hemp leaf extracts on lipopolysaccharide-induced inflammation

As shown in Figure 1B, compared with the control group, LPS (10, 50, and 100 μg/mL) did not cause damage to MDCK cells (*p* > 0.05), and LPS at 200 μg/mL causing the most obvious damage to MDCK cells was selected as the best concentration to induce inflammation in MDCK cells with statistical significance (*p* < 0.05). As shown in Figure 1D, the cell viability of the control group was higher than that of the LPS group, indicating that LPS (200 μg/mL) successfully induced MDCK cell damage. Except for the LPS + c12 group, the cell viabilities of all LPS + hemp leaf extract groups were significantly higher than that of the LPS group but lower than that of the control group, while cell viabilities of the LPS + D129 and LPS + c7 groups were the highest. The results showed that 12 hemp leaf extracts at 0.05 mg/mL have different protective effects on LPS-induced inflammatory damage in MDCK cells, and the leaves of germplasms D129 and c7 had the best anti-inflammatory effect.

**FIGURE 2:**
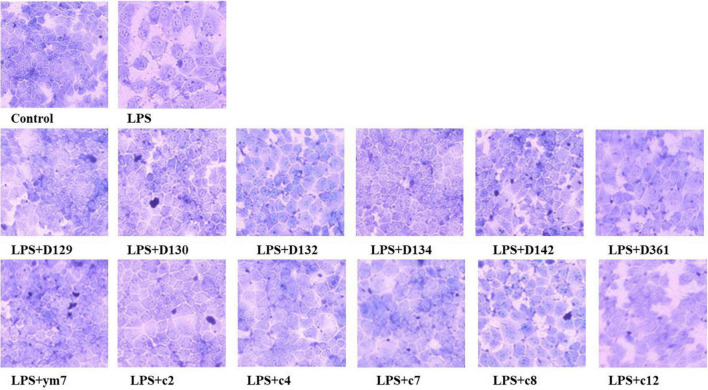
The protective effect of hemp leaf extracts on the morphology of MDCK cells with LPS-induced inflammation. Data are expressed as the mean ± SD (*n* = 3).

The morphologies of MDCK cells treated with hemp leaf extracts and LPS-induced inflammation are shown in Figure 2. The MDCK cells of the control group had no change in morphology and with clear contours. The cells treated by LPS were enlarged, with increased intercellular space, and intercellular wiredrawing occurred, which affected the normal growth and adhesion of MDCK cells. To observe the protective effect of the extracts, MDCK cells were pre-treated with 0.05 mg/mL hemp leaf extracts for 4 h and then exposed to LPS to induce a slight inflammatory insult. Compared with the LPS group, the intercellular space and cell morphology of MDCK cells pre-treated by hemp leaf extracts were more similar to the control. Even though the cell morphology images of groups LPS + c2, LPS + c4, and LPS + c12 showed the cell space slightly larger than that of normal cells, it is still smaller than that of the LPS group. The results indicated that all 12 hemp leaf extracts had protective effects on LPS-induced inflammation in MDCK cells through reducing cell structural changes and damage.

### Hemp leaf extracts decreased membrane damage of lipopolysaccharide-induced Matin-Darby canine kidney cells

**FIGURE 3:**
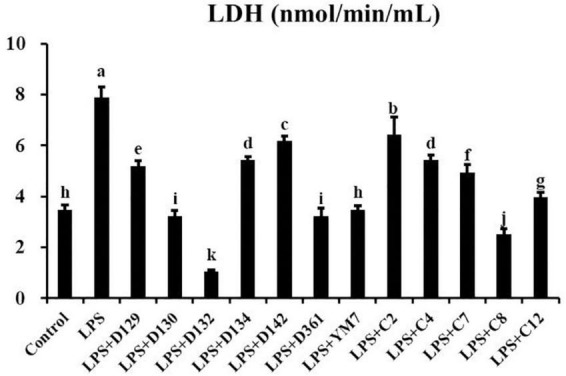
The levels of LDH released by MDCK cells treated with hemp leaf extracts and LPS-induced inflammation. Data are expressed as the mean ± SD (*n* = 3). The superscript letters (a–k) indicated statistical significance at *p* < 0.05.

When cell membranes were damaged, the release of LDH increased. As shown in Figure 3, compared with the control group, LDH concentration in the supernatant of cell suspension increased significantly after being treated by LPS (200 μg/mL) for 12 h, indicating that the cell membrane was damaged. MDCK cells were pre-treated with hemp leaf extracts at 0.05 mg/mL for 4 h followed by treatment with LPS for 12 h. The concentrations of LDH in the supernatants of cells treated with extracts were significantly lower than that of the group treated by LPS alone. In addition, the LDH concentrations of D130, D132, D361, and c8-treated groups were significantly lower than the control. The results showed that the 12 hemp leaf extracts have certain protective effects on the membrane integrity of LPS-induced inflammatory MDCK cells. Among all the germplasms, D130, D132, D361, and c8 had better effects on preventing cell membrane injury or damage.

### Hemp leaf extracts reduced the secretion of cytokines in lipopolysaccharide-induced Matin-Darby canine kidney cells

**FIGURE 4:**
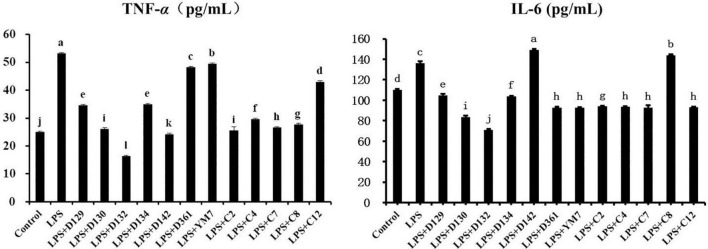
The levels of pro-inflammatory cytokines secreted MDCK cells treated with hemp leaf extracts and LPS-induced inflammation. Data are expressed as the mean ± SD (*n* = 3). The superscript letters (a–l; a–j) indicated statistical significance at *p* < 0.05.

The levels of TNF-α and IL-6 inflammatory factors released into the supernatants of cell suspensions were measured using the ELISAs kit to explore whether the reduction of MDCK cell inflammation by hemp leaf extracts was related to the inhibition of inflammatory factor secretion. As shown in Figure 4, TNF-α concentration was increased in the LPS group compared to the control group (*p* < 0.05). The concentrations of TNF-α in the hemp leaf extract-treated groups were lower than those in the LPS group, with the LPS + D123 and LPS + D142 groups even lower than the control group (*p* < 0.05). These results indicated that 12 hemp leaf extracts at 0.05 mg/mL could significantly reduce the cell secretion of TNF-α, especially the extracts from the germplasms D132 and D142. TNF-α, one of the most abundant cytokines released in patients with hyperinflammatory contexts (i.e., severe COVID-19), appears to prevent the formation of germinal center, resulting in not enough B cells capable of producing high-quality antibody responses, reducing the immune response which predisposes the host to infections (26). Thus, developments of cytokine inhibitors, especially TNF inhibitors, from natural products, may be an important means of regulating immune response.

**FIGURE 5:**
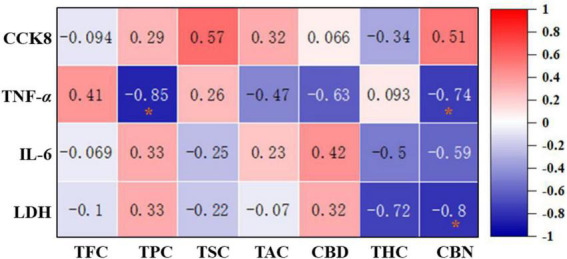
Correlation heat map between the phytochemicals and anti-inflammatory effects of 12 hemp leaves. The degree of correlation is marked in colors with different intensities, red (positive correlation) and blue (negative correlation). *Indicated statistical significance at *p* < 0.05.

For IL-6 (Figure 5), its concentration was greatly increased in the LPS group. However, LPS + D142 and LPS + c8 groups did not reduce the MDCK cell inflammation by inhibiting the release of IL-6 but triggered a sharp rise of IL-6 than LPS. Other hemp leaf extract groups significantly reduced the release of IL-6, indicating that the addition of hemp leaf extracts could reduce MDCK cell inflammation by suppressing the secretion of IL-6, especially the extracts from the germplasms D129, D130, D132, and D134.

### Heatmap correlation and clustering analysis

**FIGURE 6:**
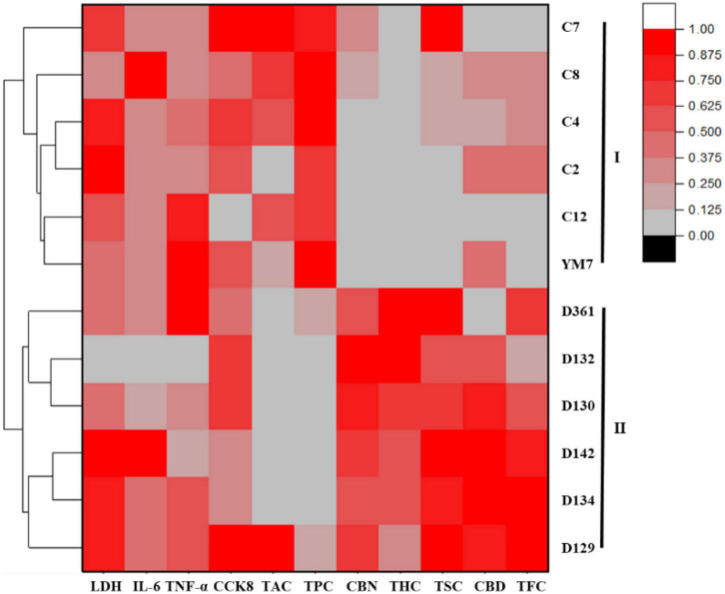
Hierarchical clustering heat map of the 12 hemp leaves, with the degree of differences marked with red and gray.

In order to evaluate the correlation between the main phytochemicals and anti-inflammatory activities of different hemp leaves, a correlation analysis was performed. As shown in Figure 6, TPC and CBN were negatively correlated with the TNF-α level (*r* = −0.85 and −0.74, respectively), suggesting that the phenolics and CBN in the hemp leaves might be the main contributors to their anti-inflammatory activities by inhibiting TNF-α secretion. CBN was negatively correlated with the level of LDH (*r* = –0.8), indicating that CBN in the hemp leaves might be the main contributors to their anti-inflammatory activities by inhibiting LDH secretion. Overall, these results clearly indicated that phenolics and CBN of hemp leaves were significantly contributed to their anti-inflammatory activities by inhibiting TNF-α and LDH secretion. Indeed, some phytochemicals in hemp and other plants were reported to exhibit anti-inflammatory activities, which provide a useful support for the better use of natural products in functional foods and nutraceuticals. For example, previous studies have shown that phenolics present in many plants with anti-inflammatory properties, such as *Zingiber officinale* (ginger) (27), *Curcumma longa* (turmeric) (27), *Pomegranate peel* (28), and *Rosmarinus offininalis* (rosemary) (27). *C. japonicum* flavonoids and saponins significantly inhibited IL-6 secretion and NO production in LPS-induced RAW 264.7 cells (29). Furthermore, CBN, a degradation product of THC, was reported to inhibit pro-inflammatory cytokine, IL-2 (30). In addition, CBD and THC were reported to exhibit significant anti-inflammatory activities (31). In particular, some previous studies demonstrated that CBD prevented LPS-induced microglial inflammation by inhibiting ROS/NF-κB-dependent signaling and glucose consumption (32) and ameliorated arthritis by targeting synovial fibroblasts (33). Also, some previous studies demonstrated that various parts of hemp plant exhibited good anti-inflammatory effects in different *in vitro* and *in vivo* models. For example, hemp inflorescence extracts substantially reduced IL-6 levels in an alveolar epithelial (A549) cell line (34), and the aqueous extract of hemp roots exhibited good anti-inflammatory effects in mice by reducing the migration of inflammatory cells (35). Anti-inflammatory natural products are regarded as important alternative treating sources for inflammatory-related disorders (36). Thus, hemp leaves with high anti-inflammatory effects may begood sources of functional ingredients against inflammatory-related disorders.

Hemp germplasm is frequently dioecious, so, is easily cross-fertilized (37). Therefore, it is vital to establish methods for distinguishing hemp germplasm from different regions of China. As the results shown in Figure 6, the hemp leaves of 12 germplasms from 2 regions of China could be divided into two groups based on their main phytochemicals and anti-inflammatory activity. The first group included six germplasms (D129, D130, D132, D134, D142, and D361) from Shanxi, and the second group consisted of six germplasms (Ym7, c2, c4, c7, c8, and c12) from Hunan. The results showed that there were significant regional differences in the contents of bioactive components and anti-inflammatory activities in the studied hemp leaves, indicating the genetic distribution of hemp resources is roughly related to geographical locations without regional hybridity. One possible reason was that the overall distribution of native hemp germplasms in China shows a geographical pattern and the northern germplasms differ from the southern germplasms to a certain extent. Other possible reasons include climates, growth conditions, soil patterns, and cultivation practices of different regions. Particularly, some hemp leaves from Shanxi Province performed better in anti-inflammatory activity than Ym7, so, it is suggested that these germplasms from Shanxi can be good alternatives to Ym7. The present study indicated that the content of TPC and CBN might be used to distinguish different germplasms in major cultivating regions. As a potential valuable source of functional foods and nutraceuticals, hemp leave is predicted to be expanded in the upcoming years as demand grows, with enormous social and economic implications (38). In the future, the scientific community should aim at discovering more hemp germplasms rich in bioactive phytochemicals, and the legislative and regulatory departments should aim at encouraging adequate clinical research to prove the safety of hemp-derived products.

## Conclusion

The current findings demonstrated that the contents of various phytochemicals in hemp leaves were generally descending from TSC > TAC > TPC > TFC > cannabinoids. In addition, hemp leaves (0.05 mg/mL), especially hemp leaves cultivated in Shanxi (D129, D130, D132, D134, D142, and D361), reduced the release of pro-inflammatory cytokines (TNF-α), reduced LDH levels, and inhibited the cell morphological changes and the membrane damage of LPS-induced inflammatory MDCK cells. Furthermore, correlation analysis indicated phenolics and CBN might be the leading contributors to protecting the MDCK cells from LPS-induced inflammation. Meanwhile, hierarchical clustering indicated hemp leaves from Shanxi performed a better anti-inflammatory activity than the common cultivar Ym7. Thus, it is suggested that these germplasms from Shanxi can be good alternatives to Ym7. Overall, the present study provides scientific evidence for the anti-inflammatory potential of hemp leaves, which can be used in nutraceuticals and functional foods.

## Data availability statement

The raw data supporting the conclusions of this article will be made available by the authors, without undue reservation.

## Author contributions

YL and R-YG conceived the project. YL and HC performed the experiments. YL analyzed the data and wrote the draft. A-PX, L-LL, HC, KK, H-YL, D-TW, H-BL, and R-YG revised and edited the manuscript. A-PX and L-LL provided materials support. R-YG provided funding support. All authors contributed to the article and approved the submitted version.

## Conflict of interest

The authors declare that the research was conducted in the absence of any commercial or financial relationships that could be construed as a potential conflict of interest.

## Publisher’s note

All claims expressed in this article are solely those of the authors and do not necessarily represent those of their affiliated organizations, or those of the publisher, the editors and the reviewers. Any product that may be evaluated in this article, or claim that may be made by its manufacturer, is not guaranteed or endorsed by the publisher.

## References

[1] Kornpointner C Sainz MA Marinovic S Haselmair-Gosch C Jamnik P Schröder K Chemical composition and antioxidant potential of *Cannabis sativa* L. roots. *Ind Crops Prod.* (2021) 165:113422. 10.1016/j.indcrop.2021.113422

[2] Vedat C Hatice T Çağatay Y Selime C. Economic viability of industrial hemp production in Turkey. *Ind Crops Prod.* (2022) 176:114354. 10.1016/j.indcrop.2021.114354

[3] Dreger M Szalata M. The effect of TIBA and NPA on shoot regeneration of *Cannabis sativa* L. epicotyl explants. *Agronomy.* (2022) 12:104. 10.3390/agronomy12010104

[4] ElSohly MA Radwan MM Gul W Chandra S Galal A. Phytochemistry of *Cannabis sativa* L. Progress in the chemistry of organic natural products. *Prog Chem Org Nat Prod.* (2017) 103:1–36. 10.1007/978-3-319-45541-9_1 28120229

[5] Pollastro F Minassi A Fresu LG. *Cannabis* phenolics and their bioactivities. *Curr Med Chem.* (2017) 25:1160–85. 10.2174/0929867324666170810164636 28799497

[6] Guo T Liu Q Hou P Li F Guo S Song W Stilbenoids and cannabinoids from the leaves of: *Cannabis sativa* L. with potential reverse cholesterol transport activity. *Food Funct.* (2018) 9:6608–17. 10.1039/c8fo01896k 30500001

[7] Liu Y Liu HY Li SH Ma W Wu DT Li HB *Cannabis sativa* bioactive compounds and their extraction, separation, purification, and identification technologies: an updated review. *Trend Anal Chem.* (2022) 149:116554. 10.1016/j.trac.2022.116554

[8] Semwogerere F Katiyatiya CLF Chikwanha OC Marufu MC Mapiye C. Bioavailability and bioefficacy of hemp by-products in ruminant meat production and preservation: a review. *Front Vet Sci.* (2020) 7:572906. 10.3389/fvets.2020.572906 33102571 PMC7545362

[9] Hartsel JA Eades J Hickory B Makriyannis A. *Cannabis sativa L. and Hemp, Nutraceuticals: Efficacy, Safety and Toxicity.* Amsterdam: Elsevier Inc (2016). p. 735–54. 10.1016/B978-0-12-802147-7.00053-X

[10] Cantele C Bertolino M Bakro F Giordano M Jędryczka M Cardenia V Antioxidant effects of hemp (*Cannabis sativa* L.) inflorescence extract in stripped linseed oil. *Antioxidants.* (2020) 9:1131. 10.3390/antiox9111131 33202647 PMC7697792

[11] Hu X Yu Q Hou K Ding X Chen Y Xie J Regulatory effects of *Ganoderma atrum* polysaccharides on LPS-induced inflammatory macrophages model and intestinal-like Caco-2/macrophages co-culture inflammation model. *Food Chem Toxicol.* (2020) 140:111321. 10.1016/j.fct.2020.111321 32289334

[12] Innes JK Calder PC. Omega-6 fatty acids and inflammation. *Prostaglandins Leukot Essent Fatty Acids.* (2018) 132:41–8. 10.1016/j.plefa.2018.03.004 29610056

[13] Li CR Yang LX Guo ZF Yang H Zhang Y Wang YM LC-MS-based untargeted metabolomics reveals chemical differences of *Cannabis* leaves from different regions of China. *Ind Crops Prod.* (2022) 176:114411. 10.1016/j.indcrop.2021.114411

[14] Xu H Zhan L Zhang L. Comparison of microwave-assisted and heat reflux extraction techniques for the extraction of ten major compounds from Zibu Piyin recipe using ultra high performance liquid chromatography with tandem mass spectrometry. *J Sep Sci.* (2016) 39:1009–15. 10.1002/jssc.201501033 26749162

[15] Wang M Wang YH Avula B Radwan MM Khan IA. Development and validation of an UHPSFC-DAD/MS method for the qualitative and quantitative determination of major cannabinoids in *Cannabis* plant extracts and products. *Planta Med.* (2016) 82:82–A28. 10.1055/s-0036-1578643

[16] Ksouda G Hajji M Sellimi S Merlier F Falcimaigne-Cordin A Nasri M A systematic comparison of 25 Tunisian plant species based on oil and phenolic contents, fatty acid composition and antioxidant activity. *Ind Crops Prod.* (2018) 123:768–78. 10.1016/j.indcrop.2018.07.008

[17] Bajalan I Mohammadi M Alaei M Pirbalouti AG. Total phenolic and flavonoid contents and antioxidant activity of extracts from different populations of lavandin. *Ind Crops Prod.* (2016) 87:255–60. 10.1016/j.indcrop.2016.04.059

[18] Pan F Su TJ Cai SM Wu W. Fungal endophyte-derived *Fritillaria unibracteata* var. wabuensis: diversity, antioxidant capacities in vitro and relations to phenolic, flavonoid or saponin compounds. *Sci. Rep.* (2017) 7:42008. 10.1038/srep42008 28165019 PMC5292746

[19] Ghane SG Attar UA Yadav PB Lekhak MM. Antioxidant, anti-diabetic, acetylcholinesterase inhibitory potential and estimation of alkaloids (lycorine and galanthamine) from *Crinum* species: an important source of anticancer and anti-Alzheimer drug. *Ind Crops Prod.* (2018) 125:168–77. 10.1016/j.indcrop.2018.08.087

[20] Zhu H Guo D Zang H Hanaor DAH Yu S Schmidt F Enhancement of hydroxyapatite dissolution through structure modification by Krypton ion irradiation. *J Mater Sci Technol.* (2020) 38:148–58. 10.1016/j.jmst.2019.03.048

[21] EyeQue Corporation. *Product Manual.* Newark, CA: EyeQue Corporation (2000). p. 1–5.

[22] Pellati F Borgonetti V Brighenti V Biagi M Benvenuti S Corsi L *Cannabis sativa* L. and nonpsychoactive cannabinoids: their chemistry and role against oxidative stress, inflammation, and cancer. *Biomed Res Int.* (2018) 20:1691428. 10.1155/2018/1691428 30627539 PMC6304621

[23] Deng G Yang M Zhao K Yang Y Huang X Cheng X The complete chloroplast genome of *Cannabis sativa* L. variety Yunma 7. *Mitochondrial DNA B Resour.* (2021) 6:531–2. 10.1080/23802359.2021.1873709 33628916 PMC7889209

[24] Maayah ZH Takahara S Ferdaoussi M Dyck JRB. The anti-inflammatory and analgesic effects of formulated full-spectrum *Cannabis* extract in the treatment of neuropathic pain associated with multiple sclerosis. *Inflamm Res.* (2020) 69:549–58. 10.1007/s00011-020-01341-1 32239248

[25] Atalay S Jarocka-Karpowicz I Skrzydlewska E. Antioxidative and anti-inflammatory properties of cannabidiol. *Antioxidants.* (2019) 9:21. 10.3390/antiox9010021 31881765 PMC7023045

[26] Kaneko N Kuo HH Boucau J Farmer JR Allard-Chamard H Mahajan VS Loss of Bcl-6-expressing T follicular helper cells and germinal centers in COVID-19. *Cell.* (2020) 183:143.e–57.e. 10.1016/j.cell.2020.08.025 32877699 PMC7437499

[27] Ogbu CP Okagu IU Nwodo OFC. Anti-inflammatory activities of crude ethanol extract of *Combretum zenkeri* Engl. & Diels leaves. *Comp Clin Path.* (2020) 29:397–409. 10.1007/s00580-019-03072-0

[28] Liu Y Kong KW Wu DT Liu HY Li HB Zhang JR Pomegranate peel-derived punicalagin: ultrasonic-assisted extraction, purification, and its α-glucosidase inhibitory mechanism. *Food Chem.* (2022) 374:131635. 10.1016/j.foodchem.2021.131635 34823934

[29] Ma Q Jiang JG Yuan X Qiu K Zhu W. Comparative antitumor and anti-inflammatory effects of flavonoids, saponins, polysaccharides, essential oil, coumarin and alkaloids from *Cirsium japonicum* DC. *Food Chem Toxicol.* (2019) 125:422–9. 10.1016/j.fct.2019.01.020 30703393

[30] Patil AS Mahajan UB Agrawal YO Patil KR Patil CR Ojha S Plant-derived natural therapeutics targeting cannabinoid receptors in metabolic syndrome and its complications: a review. *Biomed Pharmacother.* (2020) 132:110889. 10.1016/j.biopha.2020.110889 33113429

[31] Nagarkatti P Pandey R Rieder SA Hegde VL Nagarkatti M. Cannabinoids as novel anti-inflammatory drugs. *Future Med Chem.* (2009) 1:1333–49. 10.4155/fmc.09.93 20191092 PMC2828614

[32] Dos-Santos-Pereira M Guimarães FS Del-Bel E Raisman-Vozari R Michel PP. Cannabidiol prevents LPS-induced microglial inflammation by inhibiting ROS/NF-κB-dependent signaling and glucose consumption. *Glia.* (2020) 68:561–73. 10.1002/glia.23738 31647138

[33] Lowin T Tingting R Zurmahr J Classen T Schneider M Pongratz G Cannabidiol (CBD): a killer for inflammatory rheumatoid arthritis synovial fibroblasts. *Cell Death Dis.* (2020) 11:714. 10.1038/s41419-020-02892-1 32873774 PMC7463000

[34] Anil SM Shalev N Vinayaka AC Nadarajan S Namdar D Belausov E *Cannabis* compounds exhibit anti-inflammatory activity in vitro in COVID-19-related inflammation in lung epithelial cells and pro-inflammatory activity in macrophages. *Sci Rep.* (2021) 11:1462. 10.1038/s41598-021-81049-2 33446817 PMC7809280

[35] Lima KSB Silva MEGDC Araújo TCL Silva CPDF Santos BL Ribeiro LAA *Cannabis* roots: pharmacological and toxicological studies in mice. *J Ethnopharmacol.* (2021) 271:113868. 10.1016/j.jep.2021.113868 33503453

[36] Gandhi GR Jothi G Mohana T Vasconcelos ABS Montalvão MM Hariharan G Anti-inflammatory natural products as potential therapeutic agents of rheumatoid arthritis: a systematic review. *Phytomedicine.* (2021) 93:153766. 10.1016/j.phymed.2021.153766 34624807

[37] Zhang LG Chang Y Zhang XF Guan FZ Yuan HM Yu Y Analysis of the genetic diversity of Chinese native *Cannabis sativa* L. cultivars by using ISSR and chromosome markers. *Genet Mol Res.* (2014) 13:10490–500. 10.4238/2014.december.12.10 25511032

[38] Cerino P Buonerba C Cannazza G D’Auria J Ottoni E Fulgione A A review of hemp as food and nutritional supplement. *Cannabis Cannabinoid Res.* (2021) 6:19–27. 10.1089/can.2020.0001 33614949 PMC7891210

