# Supplementary material for: Phytochemical differences of hemp (Cannabis sativa L.) leaves from different germplasms and their regulatory effects on lipopolysaccharide-induced inflammation in Matin-Darby canine kidney cell lines
Source: Front Nutr. 2022 Jul 22;9:902625. doi: 10.3389/fnut.2022.902625 (PMC9355258; doi:10.3389/fnut.2022.902625)
Supplement: Supplementary file 1 [file Data_Sheet_1.pdf]

Supplementary Material

1.1 Supplementary Figures

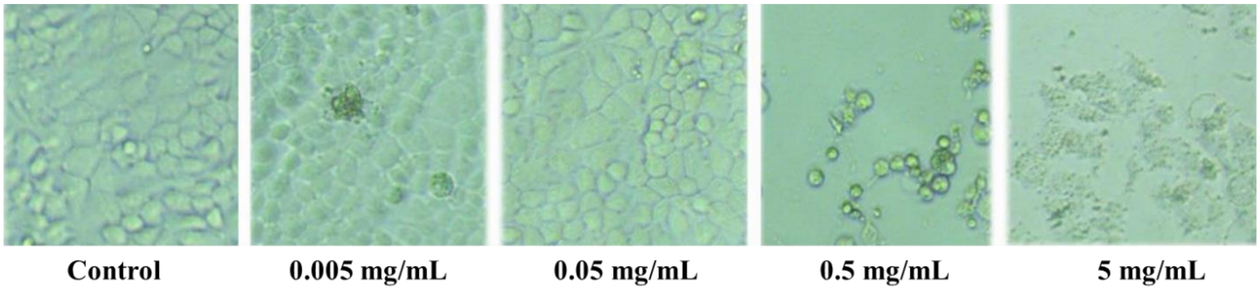

Figure S1. The effects of different concentrations of hemp leaf (Ym7) extract on MDCK cells.

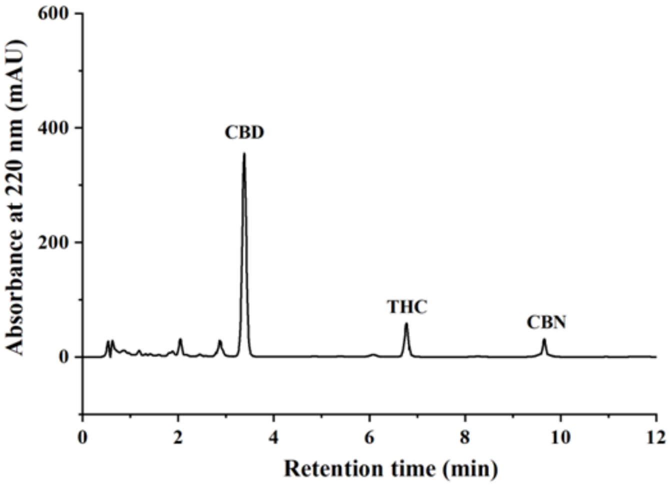

Figure S2. The HPLC chromatogram of CBD, THC, and CBN in hemp leaf. CBD, cannabidiol; THC, tetrahydrocannabinol; CBN, cannabinol.
